# What is the effect of independent medical evaluation on days on sickness benefits for long-term sick listed employees in Norway? A pragmatic randomised controlled trial, the NIME-trial

**DOI:** 10.1186/s12889-022-12800-1

**Published:** 2022-02-26

**Authors:** Silje Mæland, Tor Helge Holmås, Irene Øyeflaten, Elisabeth Husabø, Erik L. Werner, Karin Monstad

**Affiliations:** 1 Research Unit for General Practice, NORCE Norwegian Research Centre, Bergen, Norway; 2 Department of Global Public Health and Primary Care, Faculty of Medicine, University of Bergen, Bergen, Norway; 3 NORCE Norwegian Research Centre, Health and Social Sciences, Bergen, Norway; 4 Norwegian National Advisory Unit on Occupational Rehabilitation, Rauland, Norway; 5 RKBU West, NORCE Norwegian Research Centre, Bergen, Norway; 6 Department of General Practice, Institute of Health and Society, Faculty of medicine, University of Oslo, Oslo, Norway

**Keywords:** Insurance medicine, Injured workers, Sick leave, Return to work, Independent medical examination

## Abstract

**Background:**

Independent medical evaluations are used to evaluate degree and reason for work disability, uncertainty around the functional status, and/or the employee’s rehabilitation potential in several jurisdictions, but not in Norway. The main aim of this trial was to test the return to work effect of independent medical evaluation (IME) (summoning and consultation) compared to treatment as usual (TAU) in Norway, for workers who have been on continuous sick leave for 6 months.

**Methods:**

This was a pragmatic randomised controlled trial including all employees aged 18–65 years, sick-listed by their general practitioner and on full or partial sick leave for the past 26 weeks in Hordaland County, Norway in 2015/16. Trial candidates were drawn from a central register at the Norwegian Labour and Welfare Administration at 22 weeks of sick leave. Pregnant women, individuals with cancer or dementia diagnoses, those with secret address, employed by NAV or sick listed by the specialist health services were excluded. Separate regression analyses were conducted to investigate the “intention-to-treat” and “treatment on the treated” effects, using the ordinary least squares and instrumental variable methods, respectively.

**Results:**

After exemption based on predefined exclusion criteria, 5888 individuals were randomised to either IME (*n* = 2616) or TAU (*n* = 2599). The final intervention group constitutes 1698 individuals, of which 937 attended the IME consultation. No baseline differences were found between the IME and TAU group regarding gender, age, and previous sick leave. Individuals attending the IME were older than those who cancelled the appointment ((47/45), *p* = 0.006) and those who did not show up without cancelling ((47/42), *p* < 0.001). Mainly the IME physician agreed with the regular GP upon level of sick leave. In cases with different assessments, the difference tended to be towards a lower sick leave level. There were no intention to treat or treatment on the treated effect on days of sick leave after randomisation during follow up.

**Conclusions:**

Overall, the analyses showed no effect of IME on changes in sick leave for sick listed employees. This result was consistent for those who were offered an IME consultation (intention to treat) and those who undertook an IME consultation (treatment on the treated).

**Trial registration:**

ClinicalTirals.gov trial number NCT02524392 first registration 14.08.2015.

## Introduction

Independent medical evaluations (IME) are common in several jurisdictions and used to evaluate degree and reason for work disability, uncertainty around the functional status and/or the employee’s rehabilitation potential [1]. The person performing an IME is another physician than the treating physician, thus eliminating the potential bias of a long-term patient-physician relationship.

Norway has among the highest sick leave rates in Europe. Currently at 6.2% for self- and doctor-certified [2] and approximately 330 sick listed employees reach 26 weeks of continuous sickness absence every day. The governmental concern is particularly on the long-term sickness absence [3]. The general practice scheme in Norway entitles all citizens to have a regular general practitioner (GP) and most GPs are self-employed on a contract with the local authorities. They issue and manage 80% of all sickness certificates [4] and are thus a key stakeholder in sickness absence and return to work (RTW) follow-up. Although Norwegian GPs are constantly urged by the authorities to motivate and focus on RTW possibilities, the effort put into this task probably varies considerably. Patients have the option to change GP twice a year and studies have shown that competition for patients affect the GP’s sick listing behaviour [5, 6]. In Norway, graded sick leave is a priority tool in efforts to reduce sick leave [3]. GPs can grade the sick listing of a patient from 20 to 100%, and a sickness absence spell can last for maximum 1 year with full wage reimbursement (up to 61.542 Euros per year). The employer fully covers the first 16 days and then the national social security insurance system (the Norwegian Labour and Welfare Administration (NAV)) fully covers expenses from day 17 up to 1 year. Legally, the employer and employee have the main responsibility of effecting adjustments, follow-up actions and work-related activity within the first 8 weeks. In this phase and extending up to 26 weeks of sickness absence the workplace, health and insurance stakeholders does not have a formalized dialogue before NAV summon a mandatory dialogue meeting at 26 weeks. This system description constitute treatment as usual.

Several studies report conflict between multiple roles of the GP, especially between treating and caring for their patients and acting as society’s gatekeepers [7, 8]. GPs also have challenges assessing work ability and deciding grade and length of sick leave for the individual patient [7, 9]. As an initiative to try to speed up RTW, the Norwegian Ministry of Labour and Social Affairs suggested implementing IMEs for employee’s sick listed for 26 weeks. To evaluate the potential effect of IMEs on RTW before large-scale implementation, a pragmatic randomised controlled trial was planned and conducted in a county with a representative 10% sample of the whole Norwegian population [10].

To the best of our knowledge, no trial has been performed in any jurisdiction to evaluate the effect of IME. This trial is based on the hypothesis that the IME intervention would result in quicker RTW than treatment as usual (TAU) from the regular GP. Two plausible mechanisms for a positive effect of IME are i) a “notification effect”, which is found in another social insurance context [11], and ii) an effect from having the IME consultation itself. The treatment inherent in IME consists of two components in addition to TAU: i) receiving a letter with the call for an IME consultation and ii) having the IME consultation. It is possible to receive i) without ii), but not vice-versa. It is not possible to separate the effect into these two components; therefore, the effect of IME should be considered a total effect. The regular GP remained responsible for medical follow up of all sick listed employees enrolled in the study.

The main aim of this trial was to test the RTW effect of IME (summoning and consultation) compared to TAU in Norway, for workers who have been on continuous sick leave for 6 months.

## Methods

### Trial design, participants

This was a pragmatic randomised controlled trial (RCT) conducted in close collaboration with NAV. It included all employees aged 18–65 years, sick-listed (fully or partially) in 2015/16 with an International Classification of Primary Care 2nd edition (ICPC-2) diagnosis (indicating being sick listed by a GP), living in Hordaland County in Norway, and whose sickness absence spell had lasted for 26 weeks. Trial candidates were drawn from a central NAV register at 22 weeks of sick leave. Crossing 22 weeks lead to an automatic notification at the desk of a local NAV counsellor who was trained to identify eligible participants based on inclusion/exclusion criteria. The trial excluded pregnant women, individuals with secret address, NAV employees, people with International Classification of Diseases 10th edition (ICD-10) diagnoses (i.e., diagnosed and sick-listed by the specialist health services), ICPC-2 cancer or dementia diagnoses. No changes were made to the methods after trial commencement.

Representatives from NAV had an active role in all phases of setting up this pragmatic RCT. The main reason for this was that the intervention had to be temporarily implemented at NAV in Hordaland County for this trial to be conducted, while participants maintained all their legal rights. Secondly, the intervention had to be designed in such a way that NAV could implement it nationwide if the results had been in favour of the IME consultations and the politicians had decided to make IME part of NAV’s regular follow up of sick listed employees in Norway.

### Ethics and consent

The Norwegian Regional Ethical Committee (REK nr. 2015/560) assesses research proposals covered by the Health Research act and declared the project as exempt from review. The reason was that the main aim of the study was to evaluate the effect of IME on RTW. Work participation is not viewed as a health outcome by the Norwegian Health research act. As a result, the study was instead covered by the Personal Data Act. This trial was performed as part of NAV’s follow up of sick listed employees and therefore regulated by The Insurance Law Act 1997-02-28-19-§25–13 § 4 and a specific regulation for testing IME in Norway [12]. This Act authorizes NAV to summon sick listed employees receiving sickness benefits from the national social security insurance scheme to consultations. This enabled the study to be performed without informed consent from the participants. Importantly, as this was a trial and not implemented practises for all sick listed employees in Norway, there were no economical or treatment sanctions for not attending.

The study was first registered in the international register ClinicalsTirals.gov on 14.08.2015, trial number NCT02524392.

All methods were carried out in accordance with relevant guidelines and regulations.

### Interventions, randomisation

After identifying eligible participants, the NAV counsellor sent an e-mail with the participant’s ID-number, year of birth and gender to a research technician (not part of the research team conducting the trial) at NORCE for randomisation. Allocation was returned by e-mail and participants were consecutively randomised to either the IME group or the TAU group. We performed simple randomisation stratified per the 13 NAV offices in Hordaland County (1:1) where the randomisation allocation sequence was decided by use of a computer-generated randomisation list. Participants randomised to receive one IME consultation received a letter from the local NAV office with information about the new initiative and time and place for the consultation. The letter stated that the consultation was mandatory, and they were asked to prioritise the consultation. Participants randomised to TAU did not receive any information and were merely followed up as usual by their regular GP and NAV.

The RCT was carried out as part of NAV’s follow up of sick-listed employees. To test this new intervention, nine GPs were employed as IME physicians by NAV in the trial period performing the IMEs. They had offices in different parts of Hordaland County. The intervention included a consultation between the IME physician and the sick-listed employee, in a regular GP clinic, lasting up to 1 h. The focus in the consultation was assessment of previous treatment, return to work-related actions and the employee’s and the IME physician’s evaluation of work ability and RTW possibilities. The IME physician ended the consultation by writing a report (based on a predefined report scheme) to NAV on his/her findings and making a recommendation for further follow-up and sick leave grade. This report was also sent to the treating GP who assessed the need and potential for further actions. The IME physician did not have authority to change the sick leave status and according to Norwegian law the regular GPs remained responsible for the follow-up of all sick listed employees on his/her list. Thus, all judicial rights and obligations for the sick-listed employees also remained unaffected and the only factor that divided the intervention group from the TAU group was the IME summoning and, for those who met with the IME physician, the IME consultation. For simplicity, we label the treatment “IME” rather than “IME + TAU”. For full description of the IME physicians’ training program, time and form of the preparation and consultation, content of the IME report and how the IME physicians evaluated the employee’s RTW barriers and potential, please see our protocol paper [10]. No changes were made to methods after trial commencement.

### Outcomes

The primary outcome was number of days on sickness benefits, weighted and not weighted for grading, after inclusion to the trial, based on complete and objective data from NAV’s national social insurance register. There was no loss to follow-up at 7, 9, and 12 months.

Data collected throughout the project was saved in NAV’s secure online database. All directly identifiable personal data (name, social security number, address etc.) was replaced by a unique ID-number before data analyses, when data were kept in NORCE’s secure database. The main outcome, number of days on sick leave after randomisation, was measured in calendar days based on data from GPs’ sick leave certificates registered in NAV (unweighted). However, the most informative way of describing the outcome is days on sick leave after randomisation weighted with the actual sick leave grade, e.g., 1 day with 100% sick leave counts as 1 day, while 1 day with 50% sick leave counts as 0.5 day, etc.

### Sample size

Sample size calculations were based on data from Hordaland County showing that 15% of employees who had been sick-listed for 6 months in 2008 were not receiving sickness benefits at 7 months follow-up (Statistics Norway’s events database (FD-Trygd). We estimated that a 3% effect of the IME compared to the TAU group, i.e., 18% vs 15% of employees who had been sick listed for 6 months would not be sick listed at 7 months, could be discovered if 1892 individuals in two groups participate in the study. The calculations were performed in STATA 14 (StataCorp LP, USA) by the command “Power”. Statistical significance level was set to 5% with expected positive effect and power of 80%.

### Blinding

The sick-listed employees in the TAU group were not aware of their participation in the trial. The researchers assessing the outcomes (THH and KM) were blinded for intervention assignment.

### Statistical methods

For the main effect analysis, we observed mean days of sick leave after randomisation for participants in the two groups. In separate multiple linear regression analyses, we also examined effects of the intervention including adjustment for group differences in age, gender, country of birth, marital status, number of children under the age of 7 or 18, number of sick days 2 years before randomisation, calendar year and month, and geographical site indicators. We estimated the effect for all who were offered an IME consultation, i.e., an analysis adhering to the “intention-to-treat” (ITT) principle, using the ordinary least squares method. We also estimated the effect for the group who took part in the IME consultation, i.e., an effect of “treatment on the treated” (TT). In the latter analysis, randomisation status was utilized as instrument in an instrumental-variable approach. This approach solves the problem of causal inference in an RCT with partial compliance, in this case, where the group who received the IME consultation is self-selected [13].

To test for potential subgroup effects of IME, we conducted analyses of subsamples defined by gender, age, and ICPC-2 diagnosis.

## Results

**Fig. 1:**
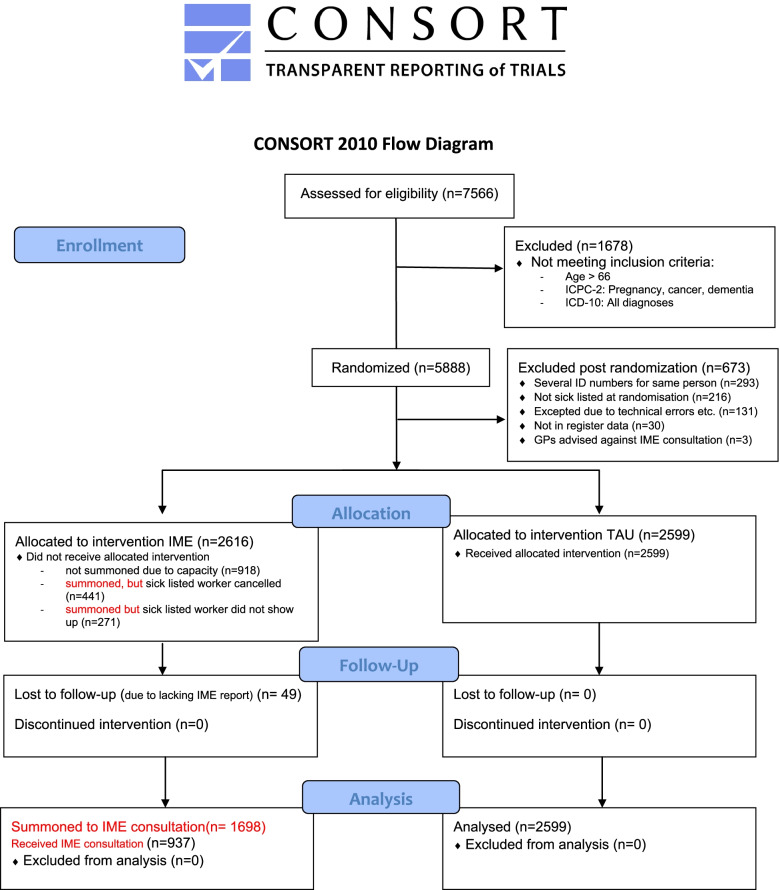
Consort flow diagram for the NIME trial

The flow diagram (Fig. 1) shows that out of 7566 long-term sick listed employees who were drawn to the trial, 1678 were exempted from the trial based on predefined exclusion criteria: The main reasons for exceptions were age above 66, pregnancy, predefined ICPC-2 diagnoses, and all ICD-10 diagnoses. Thus 5888 individuals were randomised either to the IME or TAU group. However, after randomisation, an additional 673 individuals had to be omitted from the trial. This was due to five reasons: 1) By mistake, the same individual had been drawn several times in the central register and was thus randomised several times (293 individuals: IME = 118, TAU = 175). 2) The individual was not on sick leave at the time of randomisation (216 individuals: IME = 94, TAU = 122). 3) Technical errors in connection with recruitment or new information on exclusion criteria, such as pregnancy (131 individuals: IME = 98, TAU = 33). 4) The social security number was not found in NAVs central registers, which may be due to incorrect registration at the local NAV-office (30 individuals: IME = 12, TAU = 18). 5) The treating GP or psychologist contacted the project secretary and advised against participating in the IME consultation due to severity of the medical condition (3 individuals).

Accordingly, an intervention group of 2616 individuals (IME) and a control group of 2599 individuals (TAU) remained. The distribution of the 673 individuals who had to be excluded from the trial after randomisation was not dependent on randomisation outcome, meaning that there were no systematic differences between the groups. One exception is individuals with exclusion cause number 3) (technical errors), where those randomised to the treatment group are overrepresented.

Due to IME physicians’ capacity limitations, not all of those who were randomised to the intervention group were summoned for an IME appointment, thus 918 were excluded from the IME group immediately after randomisation. Those excluded did not differ from those summoned on demographic variables such as gender, age, time for randomisation and earlier sick leave trajectory the previous 2 years. The remaining, 1698 individuals, constitute the final intervention group who received the summoning letter to an IME consultation. These individuals are included in the intention to treat (ITT) analysis.

As we can see from the flow diagram (Fig. 1), a relatively high number of those who received an invitation letter cancelled the IME appointment (*n* = 441) or failed to show-up without cancelling the appointment (*n* = 271). In 49 cases, the IME physician did not send an IME-report to NAV, and we have no information whether they attended the IME consultation or not, and therefore cannot be included in the TT effect analysis. However, they all received the summoning letter. Finally, there are 937 individuals who both received the summoning letter and who attended the IME consultation.

### Recruitment dates defining the periods of recruitment and follow up

**Fig. 2:**
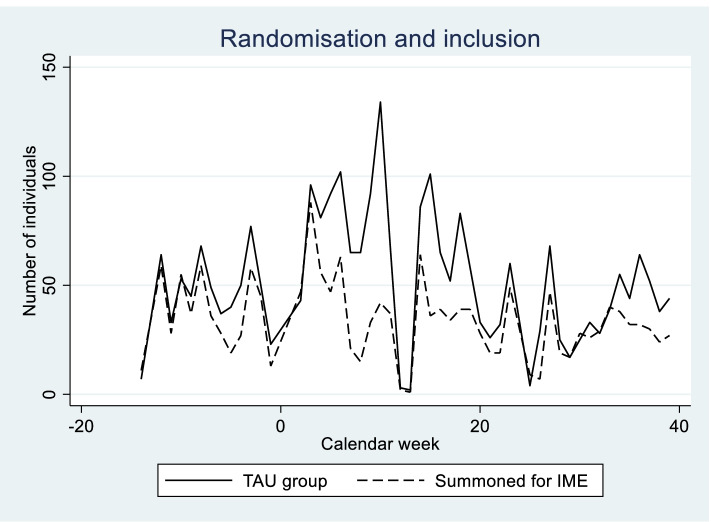
Randomisation and inclusion over time. Week 0 in the figure refers to week 52 in 2015

The predefined number of individuals were randomised to the trial between September 2015 and September 2016. After this year the inclusion was stopped. Figure 2 shows how the inclusion is distributed over time for the IME group and the TAU group. This variation is due to two factors: i) the extraction from the central NAV register was stopped prior to Christmas, Easter (week 12/13) and the summer holidays to avoid excessive waiting periods for IME consultations. ii) In some periods, especially the months of February, March and April 2016, it was difficult to obtain IME appointments for those who were randomised to IME due to capacity limitations. Thus, even though the randomisation itself was done at a 1:1 ratio, there were fewer people in the group “summoned for an IME consultation” than in the control group. In ancillary analyses, we included month of randomisation as a control variable.

There were no significant differences between participants in the IME group and the TAU group regarding gender, age, and days on sick leave before randomisation (Table 1).

**Table 1:** Background characteristics of the control- and treatment group for the intention-to-treat analysis. Mean, standard deviation (SD) and [range] of each variable

Variable	TAU^a^	IME^a^	Difference	*p*-value
Male, %	42	43	1	0.435
Age at randomisation	46 (12) [19–67]	46 (11) [18–66]	0.2	0.568
Days on sick leave the past year	34 (54) [0–273]	31 (50) [0–266]	− 2.5	0.126
Days on sick leave the past two years	18 (54) [0–388]	17 (59) [0–386]	− 0.6	0.715
Days on sick leave before randomisation	139 (23) [0–367]	140 (60) [0–327]	0.8	0.275
N	2599	1698		

^a^*TAU* treatment as usual, *IME* independent medical evaluation

### Outcomes and estimation

**Fig. 3:**
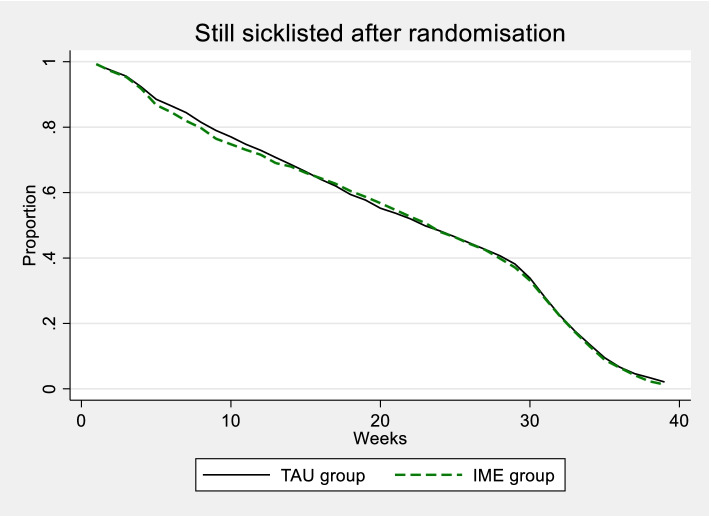
Proportion of individuals on sick leave per week after randomisation, for the control and IME groups, counting 2599 and 1698 individuals, respectively

Table 2 shows that the IME physician and the regular GP mainly agreed upon level of sick leave. Mean grade of sick leave among the participants at the time of consultation where 77.6%, whereas the IME physician recommended 11% lower sick lick leave grade (66,7). In those cases where the IME physician recommended a different level of sick leave than the regular GP, the difference tended to be towards a lower sick leave level. So far, we have only examined the effect of an IME on the total length of sick leave, over the entire period after randomisation. To give a more comprehensive picture of the development, Fig. 3 shows the development of the proportion that is still on sick leave week by week after randomisation.

**Table 2:** Sick leave level certified by the regular GP at the time of the IME consultation and recommended sick leave level from the IME physician. Mean grade of sick leave, percent

	Regular GP	IME physician	Difference
All consultations (*n* = 930)	77.6	66.7	−10.8
Same level of sick leave (n1 = 637)	78.2	78.2	0
Lower level of sick leave (n2 = 268)	79.6	40.0	−39.6
Higher level of sick leave (n3 = 25)	39.4	60.8	21.4

Among 937 participants who received an IME, there were 7 cases where the IME physician’s recommendation was not in the format of a specific sick leave level, therefore *n* = n1 + n2 + n3 *=* 930

Due to the inclusion criteria for the study, all participants were on sick leave at the time of randomisation. That means that the proportion of sick listed is 1 (one) at time zero. After about 30 weeks, the number on sick leave was strongly reduced, which is due to the fact that, by then, many had ended their period on sick leave benefit (which is maximum 1 year). This was expected since the participants had on average been sick listed for 20 weeks at randomisation. Looking at the period between these two extremes, there is a fairly even departure from sick leave over time both in the IME and TAU group. However, there is a tendency for the share on sick leave to be smaller in the IME group during the period 5–15 weeks after randomisation, while this tendency corresponds to a slightly higher proportion in a period thereafter (Fig. 3). Two months after randomisation, the difference in probability of still being on sick leave was 0.021 (ITT) and 0.040 (TT) in favour of the IME group, although not statistically significant (*p*-values of 0.101 and 0.073, respectively).

Table 3 reports the effect of IME, using two different concepts: either related to being summoned to an IME consultation, whether it is attended or not (ITT effect) or related to attending the IME consultation (TT effect). The effects are estimated without the inclusion of control variables.

**Table 3:** The effect of IME for the whole population (ITT effect) and those attending the IME consultation (TT effect)

	Days on sick leave after randomisation	*p*-value	Days on sick leave after randomisation (weighted^a^)	*p*-value
ITT effect	−1.381	0.581	−0.671	0.791
N	4297		4297	
TT effect	−3.573	0.423	−2.270	0.613
N	4248		4248	

*ITT* intention to treat (estimated by the OLS method)

*TT* treatment on the treated (estimated by the instrumental-variable method)

^a^weighted by sick leave grade

### Ancillary analyses

The inclusion of control variables (age, sex, native-born, marital status, children < 7 years, children < 18 years, number of sick days 2 years prior to randomisation, month of randomization and indicators for NAV office) did not yield any noteworthy changes in results. Changing sample selection criteria by including individuals despite pregnancy, high age or specific diagnoses did not change the sample to any great extent nor results. Neither did analyses stratified by gender, age, and sick leave diagnoses for the ITT group or the group attending the IME consultation reveal any subgroup-effects of the IME intervention.

### Harms

To avoid harm, we were open for having a dialogue with GPs or other health care professionals to contact the trial secretary about vulnerable participants. However, only three GPs contacted the secretary with concerns about the patient’s health condition resulting in an exemption from participating in the trial (See Fig. 1). No harms were reported during the trial because of the intervention.

## Discussion

The background for this intervention was to test if an IME in Norway could influence days on sickness benefits for sick listed employees at 26 weeks. However, a fairly even departure from sick leave over time was observed both in the IME and TAU group and the differences in sickness absence were not statistically significant.

### Limitations

A relevant concern regarding this analysis is the consequences of capacity problems (low IME physician capacity compared to numbers randomised to the intervention). We do not consider these problems a threat to external nor to internal validity. Table 1 shows that the control group and the IME group are balanced with respect to background characteristics. Furthermore, there are no systematic differences between those who were offered an IME consultation and those who were not, regarding gender, age, and prior sickness absence (the latter not reported here). Capacity problems reduced the sample size, but the effect estimate is so far from statistical significance that an increase in sample size to the maximum obtainable (i.e., from 4297 to 5215 participants in total) would not change the conclusion of no effect.

A major limitation to this RCT is the lack of informed consent which is an important principle in the Helsinki Declaration. However, as this was a pragmatic RCT evaluating the effect of a political initiative that could easily have been implemented nationwide without knowledge of effect, we carefully considered the potential harms and benefits for individuals and the society. Combined with the minimal risk of harm and close dialogue with ethical boards we found it justifiable to conduct the RCT without informed consent [10].

### Interpretation

One important aspect of the IMEs in this trial was that the IME physicians were GPs themselves and therefore familiar with the dilemmas involved in following up long-term sick listed employees. The background for engaging GPs as IME physicians was based on close dialogue with the Norwegian Medical Association and the general practice community in Norway in designing the trial. They clearly stated that advice had to come from someone who knew general practice. IME physicians in other jurisdictions with a different medical speciality have been criticized for lacking experience with the health problem in question and lacking skills in compensation and RTW issues [14, 15]. Our previous findings have shown that the treating GPs trusted their experienced peers acting as IME physicians in the NIME-trial and that they were positive to a second opinion that might detect their own blind spots [16]. However, our results show that they did not act in line with the IME physicians’ recommendations in 268 cases where the IME physicians recommended no or lower sick leave grade. One reason could be that the treating GPs did not find the IME-report useful and that they wanted to handpick challenging patients for peer IMEs rather than allocation being determined by randomisation [16]. Handpicking however, has been tried in a previous trial where GPs discussed sick leave issues in challenging patients with experienced insurance physicians (chief medical advisors in NAV). This intervention did not reduce sick leave grade or rate [17]. In this trial, we found that the IME physician recommended 11% lower sick leave grade than the mean grade among participants at the time of IME consultation (Table 2). However, we note that the IME consultation takes place on average 3 weeks after the regular GP ‘s most recent sickness certificate prolongation. Over time, there is a certain RTW from sickness absence, in the control group as well, as can be seen from Fig. 3. When this fact is considered, the actual difference in opinion between regular GP and IME physician would probably have been much lower than 11% if they had evaluated the patient at the same date.

One may speculate why IMEs in the Norwegian setting did not reduce sick leave rate or grade. Long-term sick leave is a complex phenomenon, influenced by multiple factors such as personal, health care system, organisational (work), legal and insurance system factors [18]. However, jurisdictions that have changed the reimbursement rate, introducing economic incentives have succeeded [19]. Several recent studies using Norwegian data conclude that sickness absence is indeed possible to influence by moderate institutional changes, whether it is closer follow-up by NAV and change of wording in the communication to the sick-listed [20], the regular GP’s use of graded sick leave [21, 22], degree of GP competition [5, 6] or GP remuneration schemes [23]. Thus, the incentives that GPs and sick listed are faced with seem to matter. An alternative to the design of this RCT could be to strengthen the role and number of chief medical advisors (CMA) in NAV. Today there are about 110 CMAs in Norway, predominantly in part time positions. They mainly advise NAV case managers on medical issues when assessment of work ability and right to social security benefits is challenging. Changing the Norwegian CMAs role to become closer to international IME physician’s role may have given a different result. However, as shown in a study from Belgium, a dysfunctional relationship between GP, occupational physicians (OPs) and insurance physicians (IPs) impeded RTW. This was explored in a Delphi process and inter-physician conflict and lack of mutual recognition were key findings calling for reciprocal knowledge and trust in a system where third-party physicians (the OP and IP) already handle sick leave [24].

### Generalizability

In this IME intervention the sick listed employees faced weak incentives, rather an offer of a second opinion. In most cases (*N* = 637), there was no difference between the regular GPs´ current sick leave grade and IME physician’s recommendation for onward sick leave grade. In general, the regular GP kept responsibility for follow-up in the context of this pragmatic RCT. One may argue that if the IME physicians´ recommended sick leave grade had overruled the regular GPs decision, as practiced in jurisdictions such as Australia, Canada, and the USA, 268 cases would have had their sick leave grade changed and we would have seen an effect of the IMEs without further austerities. This reduces the generalizability of our results beyond contexts where family doctors or regular GPs are highly autonomous, such as in Norway.

A potential positive impact of an IME is identification of insufficient follow-up for vulnerable patient groups. Data from the IME consultation reports from the current RCT, showed that men with mental sick leave diagnoses were at risk of receiving insufficient follow-up by their GP compared to the reference group, women with musculoskeletal sick leave diagnoses [25]. This exemplifies the potential need for IMEs in certain vulnerable groups for relevant action in the intersection between healthcare and work life [25]. Previous research has shown that the larger the distance (i.e., a GP will be close due to frequent contact and an insurance physician will be distant due to rare or no contact) between the patient and the physician, the easier it is to make strict sick leave decisions [26]. Allowing the IME physician to have the last word would have made the Norwegian IME more like IMEs in other jurisdictions [26]. However, it would also have introduced the most criticized aspect of IMEs, namely letting a physician who does not know the employee, his/ her health problems, and context well, make decisions for vulnerable and marginalized individuals [15, 27].

Overall, the analyses showed no effect of IME on changes in sick leave or RTW for sick listed employees. This result was consistent for those who were offered an IME consultation (ITT) and those who undertook an IME consultation (TT). The effect evaluation of IME at 26 weeks in the Norwegian context was called for and of great importance. However, if we as researchers had been free from the call and funding scheme, we would have aimed for an earlier time point for the IME. The main reasons for this are that the IME is a simple, low intensity, low-cost intervention [12] that could have been introduced at 8–12 weeks of sick leave as suggested by van Duijn et al. [28]. Further, the literature suggests that work disability becomes more complex and that psychosocial factors become gradually more important over time [29] indicating the need for more intensive and complex interventions [12] at 26 months of continuous sickness absence.

There were already around 40 measures available at NAV with undocumented effect seeking to promote work sustainability. Internationally, introducing medical experts to determine eligibility for disability benefits in claimants with impaired health is used by both social and private disability insurers. However, anecdotal evidence suggests that experts often disagree in their judgment of capacity to work when assessing the same claimant [30]. Contrary to this we found that the regular GPs and the IME physicians agreed in most cases. Barth et al. [30] went through 23 studies from 12 countries and found that medical experts only reach low to moderate reproducibility in their judgment of capacity to work. Even though the evaluation procedure was not identical between the GP and IME physician, they had the same medical specialty (general practice/family medicine). This may explain the high level of agreement and should be considered a strength of this study as reproducibility in judgment of capacity to work is called for [30].

## Data Availability

The Norwegian Labour and Welfare Administration gave administrative permissions to access data and records. Because data are owned by the Norwegian Labour and Welfare Administration and derived from their registries after an application process within the project, sharing is not allowed. No data are available for secondary analyses. The Norwegian Labour and Welfare Administration contact information: fou@nav.nonav.statistikk@nav.no / https://www.nav.no/no/nav-og-samfunn/kunnskap/data-og-forskning-pa-nav
